# Three-dimensional topography of scapular nutrient foramina

**DOI:** 10.1007/s00276-020-02441-7

**Published:** 2020-02-28

**Authors:** J. C. E. Donders, J. Prins, P. Kloen, G. J. Streekstra, P. A. Cole, R. P. Kleipool, J. G. G. Dobbe

**Affiliations:** 1 Department of Orthopedic Surgery, Amsterdam UMC, University of Amsterdam, Amsterdam Movement Sciences, Meibergdreef 9, 1105 AZ Amsterdam, The Netherlands; 2 Department of Biomedical Engineering and Physics, Amsterdam UMC, University of Amsterdam, Amsterdam Movement Sciences, Meibergdreef 9, 1105 AZ Amsterdam, The Netherlands; 3 Department of Orthopedic Surgery, University of Minnesota, Regions Hospital, St. Paul, MN USA; 4 Department of Medical Biology, Amsterdam UMC, University of Amsterdam, Amsterdam Movement Sciences, Meibergdreef 9, 1105 AZ Amsterdam, The Netherlands

**Keywords:** Scapula, Nutrient foramen, Vascularity, Three-dimensional topography, Computed tomography

## Abstract

**Purpose:**

The aim of this study is to describe the number and location of the nutrient foramina in human scapulae which can minimize blood loss during surgery.

**Methods:**

30 cadaveric scapulae were macerated to denude the skeletal tissue. The nutrient foramina of 0.51 mm and larger were identified and labeled by adhering glass beads. CT scans of these scapulae were segmented resulting in a surface model of each scapula and the location of the labeled nutrient foramina. All scapulae were scaled to the same size projecting the nutrient foramina onto one representative scapular model.

**Results:**

Average number of nutrient foramina per scapula was 5.3 (0–10). The most common location was in the supraspinous fossa (29.7%). On the costal surface of the scapula, most nutrient foramina were found directly inferior to the suprascapular notch. On the posterior surface, the nutrient foramina were identified under the spine of the scapula in a somewhat similar fashion as those on the costal surface. Nutrient foramina were least present in the peri-glenoid area.

**Conclusion:**

Ninety percent of scapulae have more than one nutrient foramen. They are located in specific areas, on both the posterior and costal surface.

## Introduction

A nutrient artery enters the bone through a nutrient foramen, which is the principal pathway of a blood vessel into the bone. The nutrient arteries that enter the foramina can be lacerated due to fracture or surgical exposure, causing blood loss. The blood supply of the bone is important in the formation of callus around the fracture site [25]. During operative repair of scapula fractures, excessive bleeding can occur from these nutrient vessels once damaged.

As there is an increased interest in operative treatment of scapular fractures, it is useful to know whether there are anatomical patterns for nutrient foramina. Detailed topographical knowledge of these nutrient foramina is advantageous to minimize intraoperative bleeding [25, 34].

Numerous authors have documented location and number of nutrient foramina in various bones of the human skeleton [3–6, 8–12, 14–25, 27, 28, 30–32, 35]. In terms of comparative anatomy in humans, the ilium is comparable to the scapula. The nutrient foramina of the ilium have been well described [8]. Most studies on the risks of scapular surgery in relation to iatrogenic neurovascular damage have pertained to the suprascapular nerve and artery [34]. The aim of this study is to identify regions where scapular nutrient foramina are likely to occur using three-dimensional (3D) computed tomography (CT) of human cadaveric scapulae, offering better surgical guidance during operative exposure and treatment of scapular pathology.

## Methods

### Specimens

A total of thirty cadaver scapulae (ten pairs and ten single scapulae, male/female ratio 12/15, with 3 unknown), with a mean age of 76 years (range 41–91 years) at the time of death were obtained from the Department of Medical Biology, section Clinical Anatomy and Embryology of the Amsterdam University Medical Centers (location AMC), University of Amsterdam, The Netherlands.

### Dissection and maceration

Each scapula was dissected from the cadaver leaving the surrounding soft tissues intact. The proximal humerus was removed leaving the glenoid intact. After sharply dissecting all muscles and tendons from the scapula, residual soft tissue was macerated using warm water (80 °C) for 40 h, based on an established protocol from our Anatomy Department. This removed residual soft tissues without damaging the bone surface itself.

### Placement of markers for identification and localization of the nutrient foramen

Once fully cleaned, the bone surfaces of all scapulae were scrutinized on the posterior and costal surfaces and borders to assess number and location of the nutrient foramina. Based on methods widely used in literature [4, 6, 21, 22, 24, 35], only those entry points that admitted the tip of a 24-gauge wire (0.51 mm) were considered nutrient foramina. Hence, nutrient foramen of 0.51 mm and larger were included in this study. To locate the nutrient foramina on 3D CT scans, a 2-mm glass bead was glued on each nutrient foramen (Simplant, Dentsply, Zoetermeer, The Netherlands). The glass beads show up as highly intense small opacities in 3D CT scans which enable automatic position detection, hence identifying nutrient foramina locations [1]. Three additional 2-mm glass beads served as anatomical landmarks and were glued onto the anterolateral corner of the acromion (A), the medial border of the spine of the scapula and the distal tip of the inferior angle. These three landmarks define a frame of reference that is approximately in the coronal plane and is used to identify regions where nutrient foramen occur [33].

### Image acquisition and data analysis

**Fig. 1:**
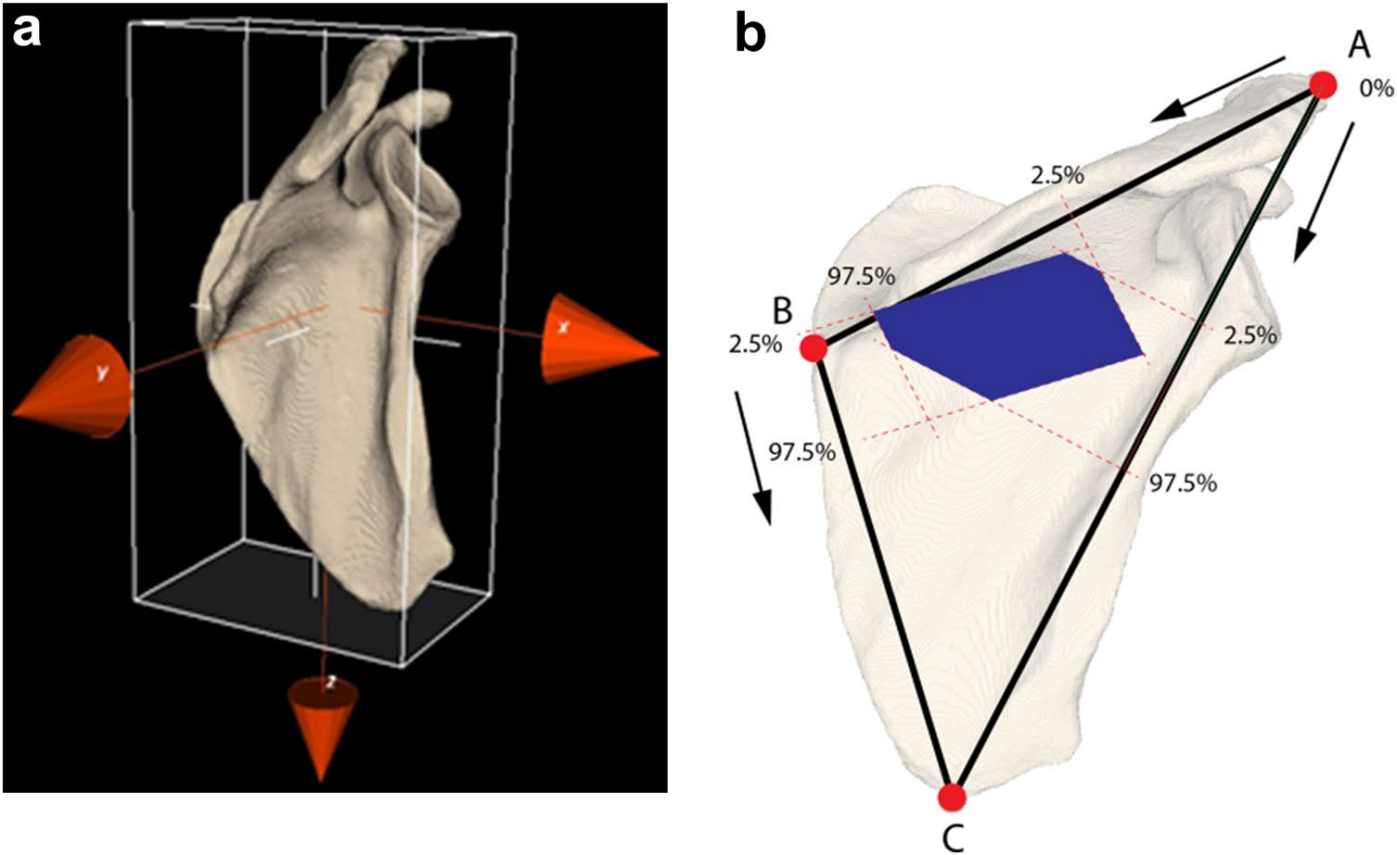
**a** Segmented scapula model showing a local coordinate system based on the axes of inertia (red *X*, *Y*, and *Z* axes). All scapulae are aligned by their local coordinate system, and scaled anisotropically by the dimensions (height, width, thickness) of the "bounding box" (white girder) of each scapula. **b** Nutrient foramina locations are projected on the lines between AB, AC and BC and provide the 2.5% and 97.5% percentile, and hence the 95% confidence range where nutrient foramina are likely to occur. By back projecting these ranges perpendicular to the respective lines (red dashed lines) a region (blue) is identified were nutrient foramina are likely to occur. A: anterolateral corner of acromion, B: medial border of the spine of the scapula, and C: inferior angle of the scapula

All 30 scapulae were individually scanned with a Brilliance 64 CT scanner (Philips Healthcare, Best, The Netherlands) (120 kV, 150 mAs, slice thickness 0.9 mm, slice increment 0.45 mm). All attached glass beads were automatically detected from these scans. To compare scapulae of different sizes, we first segmented each scapula from its CT scan using custom-made software designed by one of the authors (JD) [7]. This provided a virtual surface model (polygon mesh) for each scapula. CT scans of left scapulae were mirrored to a virtual right scapula to be able to evaluate the topography of all scapulae in the same fashion. Then, the axes of inertia of each virtual scapula model were determined, which was used to align all scapulae (Fig. 1a). After alignment, we defined the height, width and thickness for each scapula using a bounding box. This bounding box fits the scapula and enables anisotropic scaling (height, width, thickness) of all scapulae to one, arbitrarily chosen, reference scapula (Fig. 1a). All ventral and dorsal nutrient foramina positions were scaled to fit the same reference scapula and were finally projected on the respective side of that reference scapula. Based on the scapular osteology, the following topographical areas were defined; Group 1: subscapular fossa on the costal surface, Group 2: supraspinous fossa on the posterior surface, Group 3: infraspinous fossa on the posterior surface, and Group 4: peri-glenoid area of the scapula. Each nutrient foramen detected from the CT image was assigned to one of the four topographical areas based on its detected position.

To visualize regions where nutrient foramina are likely to occur, we drew three lines (AB, AC, BC) between the average positions of the glass beads A, B and C that identified the bony landmarks (Fig. 1b). The scaled nutrient foramina locations were subsequently projected on these lines to provide the 2.5% percentile and 97.5% percentile of the position distribution along each line and, hence, the 95% confidence range where these nutrient foramina project on the respective lines. Back projection of these three 95% confidence ranges perpendicular to the lines AB, AC and BC define the region on the reference scapula where nutrient foramina are likely to occur. This procedure can only identify regions in anterior and posterior view since the markers are defined in approximately the coronal plane.

## Results

**Fig. 2:**
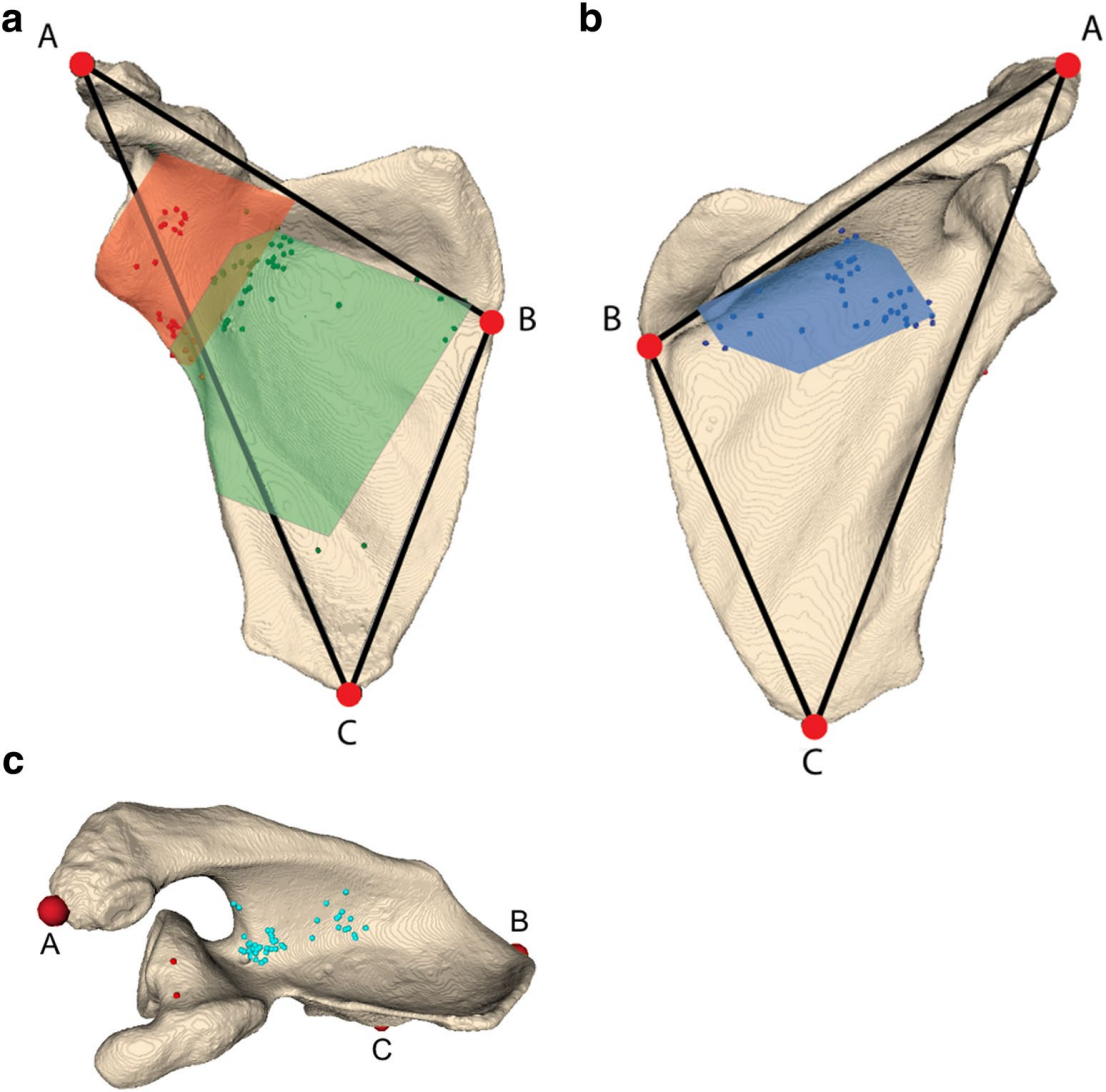
**a** Costal surface of the scapula showing 95% of the nutrient foramina on the subscapular fossa (green) and peri-glenoid area (red). A, B and C are the scapula landmark locations (see Fig. 1a). **b** Posterior surface of the scapula showing 95% of the nutrient foramina located on the infraspinous fossa (blue). A, B and C are the scapula landmark locations (see Fig. 1a). **c** Superior view of the scapula showing nutrient foramina on the supraspinous fossa (light blue). A, B and C are the scapula landmark locations (see Fig. 1a)

By visual inspection, 158 nutrient foramina were identified in 30 scapulae. On average, each scapula had 5.3 nutrient foramina (range 0–10). Only one scapula (3%) had no nutrient foramen; whereas, ninety percent had more than one nutrient foramen. The number of nutrient foramina as categorized in each of the four topographic groups is listed in Table 1. The supraspinous fossa was the location with the highest number of nutrient foramina (29.7%) of all specimens. The fewest nutrient foramina were found in the peri-glenoid area (17.7%). On the costal surface, most nutrient foramina were found inferior to the suprascapular notch and adjacent to the glenoid (Fig. 2a). There were 29 (18.4%) nutrient foramina encountered around the medial border and 2 (1.3%) around the inferior angle. On the posterior surface, the nutrient foramina were identified under the spine of the scapula in a somewhat similar fashion as those on the costal surface just proximal and medial to the collum scapulae (Fig. 2b). From a superior view, there were two clusters of nutrient foramina in the supraspinous fossa, one in the spinoglenoid notch and one more proximal in the hollow of the supraspinous fossa approximately in the middle of the spine of the scapula (Fig. 2c).

**Table 1:** Nutrient foramina (NF) per topographical area

Topographical area	Number of NF (*n* = 158)	Number of scapulae (*n* = 30)	Average number of NF per scapula (range)
Subscapular fossa	41 (25.9%)	25 (83.3%)	1.6 (0–4)
Supraspinous fossa	47 (29.7%)	28 (93.3%)	1.7 (0–3)
Infraspinous fossa	42 (26.6%)	26 (86.7%)	1.6 (0–3)
Peri-glenoid	28 (17.7%)	16 (53.3%)	1.8 (0–2)

Total NF per scapula 5.3 (0–10)

The position of the nutrient foramina in relation to the lines between landmarks A, B, and C is shown in Table 2 and Fig. 2. With the landmark frame made from the lines AB, BC, and AC, and the perpendicular position of the nutrient foramina to these lines, three topographical areas could be quantified. Data of the location of the nutrient foramina were not normally distributed (Kolmogorov–Smirnov and Shapiro Wilkinson test; *p* < 0.05); therefore, the topographical areas were presented with 2.5th and 97.5th percentiles and 95% confidence range (Fig. 1b).

**Table 2:** Topographical areas projected on the lines between the references points, expressed in % of the respective line lengths (median, 95% central range)

Topographical area	Distance in %	Distance in %	Distance in %
	AB line^a^	AC line^b^	BC line^c^
Subscapular fossa	54.0 (46.5–94.9)	37.1 (33.0–79.2)	8.7 (− 1.6 to 64.7)
Supraspinous fossa	44.3 (37.8–62.4)	29.0 (24.8–38.0)	− 2.1 (− 6.1 to 1.7)
Infraspinous fossa	57.7 (49.7–87.8)	40.2 (33.3–53.9)	6.7 (− 4.1 to 20.4)
Peri-glenoid	40.6 (23.5–52.4)	37.9 (15.8–46.7)	28.7 (− 10.2 to 37.7)

^a^AB line—distance between the anterolateral acromion (0%) and the medial border spina (100%)

^b^AC line—distance between the anterolateral acromion (0%) and the inferior scapular angle (100%)

^c^BC line—distance between the medial border spina (0%) and the inferior scapular angle (100%)

The topographical area of nutrient foramina on the subscapular fossa (costal surface) is found at 54.0% (range 46.5–94.9%) of the AB line (Fig. 2a—green). The topographical area of nutrient foramina around the glenoid (peri-glenoid area) is identified at 40.6% (range 23.5–52.4%) of the AC line (Fig. 2a—red). The topographical area of nutrient foramina on the infraspinous fossa (posterior surface) is located at 57.7% (range 49.7–87.8%) of the AB line (Fig 2b—blue)

## Discussion

This study documents the location and number of nutrient foramina (diameter ≥ 0.51 mm) in human cadaveric scapulae. After mapping the foramina onto a reference scapula, clusters were identified in specific areas. Most nutrient foramina were located in the central area of the spine of the scapula, on the infraspinous and supraspinous fossa, and on the costal surface, as well as adjacent to the glenoid. Interestingly, the number of nutrient foramina in specified areas differed between specimens.

The arterial blood supply to the scapula is not well described. Numerous authors have reported on the nutrient foramina of bones [3–6, 8–25, 27, 28, 30–32, 35]. Except for the clavicle [8] and ilium [17], these reports only describe tubular or long bones. The long bones have one major arterial supply which is the nutrient artery that reaches the bone through the nutrient foramen. The scapula (similar to the clavicle and ilium) is a flat bone which vascularization comes from superficial periosteal arterioles. However, the spine of the scapula and glenoid are the thickest, most voluminous parts of the scapula and we expected that nutrient arteries also supply this area. This last assumption is in line with our findings.

Absence of nutrient foramina is not uncommon in long bones [18, 21]. We also observed this in one of the scapulae. In such cases, it is likely that the bone is vascularized by superficial periosteal arterioles and other small vessels only.

Most reports on the arterial supply of the scapula are from the plastic surgery literature and focus on the vascular distribution to the inferior angle as it relates to pedicle grafting of a scapular graft [26]. Additional literature focuses on the blood vessels around the scapula that are at risk during a posterior approach to the scapula itself [8, 34]. One of these studies identified a relationship of the circumflex scapular artery to anatomical landmarks of the scapula and defined a high-risk area for the ascending branch of the circumflex scapular artery and its anastomosis with the suprascapular artery that passes through the suprascapular notch [34]. They studied the vessels up to their entry in the subscapular muscle but did not discuss any vessels penetrating the bone. In a recent study by Singh et al., which also used macerated cadaveric scapulae, the openings and courses of intraosseous vascular tunnels were reported. However, their location and number were not quantified [29].

Our data showed a consistent presence of nutrient foramina around the glenoid (Fig. 2). This area is of great importance as Armitage et al. found that 68% of the scapular fractures involve the inferior aspect of the collum scapulae [2].

In this study, we used a 24-gauge wire (0.51 mm) to identify a nutrient foramen as is common in applicable literature. Although the cut-off for a nutrient foramen was taken as 0.51 mm (24 gauge), the actual nutrient vessel diameter may be smaller than 0.51 mm. Therefore, smaller vessels could have been missed, and this may be likely in the bones in which few or no foramina were detected. Numerous small holes (< 0.51 mm) were indeed seen on the borders as well as around the glenoid in multiple specimens. Their small sizes suggest that these holes most likely represented attachment of muscle tendons, ligaments or small vessels. It is hard to predict what vessel size can be a risk for problematic intra-operative bleeding during surgery, but it is clear from the experience of operating surgeons that nutrient foramina bleed profusely in some of the areas that we identified. Second, we do not know whether these scapulae had sustained a fracture in the past or whether there were associated bone diseases. Visual inspection, however, did not show any anomalies.

The strengths of this investigation are that this is the first report describing the locations of the nutrient foramina in human scapulae. Also, rather than simply reporting number and location on various sizes and shapes of cadaveric bones like all other studies did so far, we scaled all cadaveric bones to one size with easily recognizable landmarks making clinical application much easier.

In conclusion, this study provides information of the location and number of nutrient foramina of the scapula that will help the operating surgeon in preventing excessive blood loss during scapular fracture repair.
